# Molecular detection and characterisation of *Toxoplasma gondii* in eastern barred bandicoots (*Perameles gunnii*) in Victoria, Australia

**DOI:** 10.1016/j.ijppaw.2025.101071

**Published:** 2025-04-11

**Authors:** K.L.D. Tharaka D. Liyanage, Michael Lynch, Oluwadamilola S. Omotainse, Chunlei Su, Jasmin Hufschmid, Abdul Jabbar

**Affiliations:** a Department of Veterinary Biosciences, Melbourne Veterinary School, Faculty of Science, The University of Melbourne, Werribee, Victoria, 3030, Australia; b Zoos Victoria, Parkville, Victoria, 3052, Australia; c Department of Microbiology, University of Tennessee, Knoxville, USA

**Keywords:** *Toxoplasma gondii*, Quantitative polymerase chain reaction, Genotyping, Eastern barred bandicoots, Australia

## Abstract

Australian marsupials are particularly susceptible to *Toxoplasma gondii*, an introduced zoonotic protozoan parasite. Molecular diagnostic methods are a highly specific approach for the detection of parasitic infections such as *T. gondii*. Importantly, molecular methods are useful for the characterisation of *T. gondii* to understand the genetic diversity of the parasite. The eastern barred bandicoot (*Perameles gunnii*) is a small native marsupial species classified as Critically Endangered. Although the species has previously been described as highly susceptible to infection with *T. gondii*, there is currently no information on the genotypes occurring in this species. This study employed qPCR for the detection of *T. gondii* in opportunistically obtained tissue samples from eastern barred bandicoot carcasses (*n* = 113) from Victoria, followed by determination of genotype using a DNA sequence-based virtual restriction fragment length polymorphism (RFLP) method. Overall, 19.5 % of the samples were positive for *T. gondii* using qPCR. The RFLP analysis revealed the dominance of *T. gondii* type II while a type II-like genotype was found in two isolates. This is the first study to provide information on prevalent genotypes of *T. gondii* in eastern barred bandicoots. Epidemiological studies of definitive and intermediate hosts, including further genotyping, are recommended to better understand *T. gondii* epidemiology for the successful recovery of eastern barred bandicoots in Australia.

## Highlights

• Eastern barred bandicoots (*Perameles gunnii*) were tested for *Toxoplasma gondii.*
• 19.5 % (22/113) of animals were positive for *T. gondii* using qPCR.
• Genotyping analysis revealed type II and type II-like genotypes of *T. gondii.*
• This is the first report of molecular characterisation of *T. gondii* in bandicoots.
• Toxoplasmosis may threaten the survival of this critically endangered marsupial.

## Graphical abstract

**Figure:**
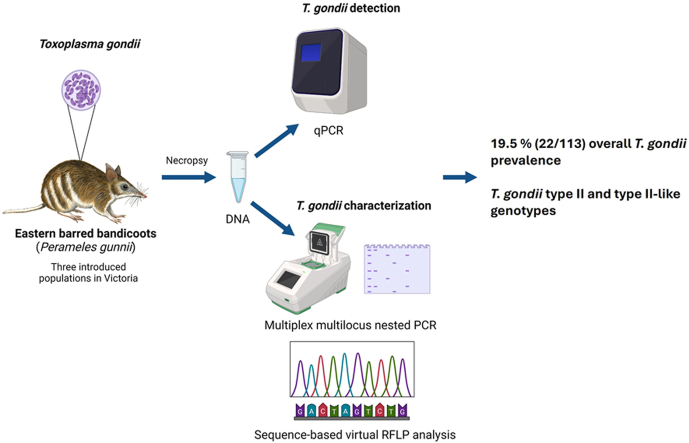

## 1 Introduction

*Toxoplasma gondii* is an emerging global zoonotic pathogen for wildlife health (Chomel et al., 2007; Tenter et al., 2000). Feline species act as the definitive host for this parasite (Dubey, 2004) and will excrete millions of environmentally resistant oocysts in their faeces after sexual reproduction of the parasite within their gastrointestinal tract (Frenkel et al., 1970, 1975). A wide range of mammalian and avian intermediate hosts may subsequently be infected by ingesting infected oocysts through contaminated food, water and soil (Dubey and Jones, 2008). Asexual reproduction occurs in intermediate hosts by the rapid multiplication of the tachyzoite stage within intestinal cells, followed by systemic circulation and then transformation into dormant bradyzoites within tissue cysts (Dubey, 2004; Tenter et al., 2000). Ingestion of bradyzoite-containing tissue cysts and vertical transmission are additional sources of infection with *T. gondii* in intermediate hosts (Dubey and Jones, 2008; Tenter et al., 2000).

Toxoplasmosis is subclinical and chronic in most healthy individuals (Dubey and Jones, 2008). However, congenital anomalies and reproductive problems can occur if infected during pregnancy, and fatal toxoplasmosis has been reported in immunocompromised populations (Dubey, 2004; Montoya and Liesenfeld, 2004; Wang et al., 2017). In small ruminants, *T. gondii* is a major cause of abortion (Dubey, 2004; Innes, 2010). Generally, Australian marsupials are thought to have a higher susceptibility to *T. gondii* than eutherian mammals (Dubey et al., 2021) due to their geographical isolation and absence of feline species during the evolutionary periods (Johnson et al., 1988). Cats (*Felis catus*) were introduced to Australia nearly 250 years ago by early European settlers and subsequently became widespread throughout the mainland and many offshore islands (Dickman, 1996; Johnson et al., 1988). It has been suggested that exposure to *T. gondii* in Australian cats is widespread, with a recent study showing a total seroprevalence of exposure to *T. gondii* of 40.4 % in free-roaming cats representing four Australian states (Liyanage et al., 2024).

Infection with *T. gondii* has been associated with overt toxoplasmosis and mortalities in many Australian marsupial species and has therefore been suggested as a significant contributor to species declines (Dickman, 1996; Woinarski et al., 2011). However, the prevalence of infection and severity of clinical outcomes vary across marsupial species (Hillman et al., 2016). In some marsupial species, such as the eastern barred bandicoot (*Perameles gunnii*), it is thought that infection may result in peracute death (Obendorf and Munday, 1990).

Eastern barred bandicoots are small nocturnal marsupials once endemic to southeastern regions of Australia, including Victoria and South Australia (Cook et al., 2010; Seebeck, 1979). Habitat loss and predation by introduced foxes (*Vulpes vulpes*) and cats reduced this bandicoot population to near extinction by the late 1980s, and the mainland population was listed as Functionally Extinct in 2008 (de Milliano et al., 2016; EPBC-Act, 1999; Winnard and Coulson, 2008). In 1991, an intensive captive breeding program was initiated, and subsequently, several reintroduction attempts to the former range of eastern barred bandicoots were made with varying degrees of success (Mitchell et al., 1992; Todd et al., 2002). Nonetheless, following three decades of reintroduction and assisted colonisation attempts, the conservation status of eastern barred bandicoots on the mainland could be reclassified from Functionally Extinct to Endangered, becoming the first Australian species to upgrade its conservation status (EPBC-Act, 1999; Parrott et al., 2017). While previous work has shown susceptibility of the Tasmanian subspecies of eastern barred bandicoots to infection with *T. gondii* both in experimental and observational studies (Bettiol et al., 2000a; Obendorf and Munday, 1990), the parasite has not previously been demonstrated in their tissues using molecular methods, nor have genotypes associated with infection in the species been described.

Eastern barred bandicoots are thought to frequently succumb to infection prior to the development of IgG (Bettiol et al., 2000a). Therefore, antemortem diagnosis of infection with *T. gondii* using established serological methods such as the modified agglutination test (MAT), which only detects later-phase immunoglobulins (IgG), may be problematic. Several molecular approaches for the detection of *T. gondii* DNA have been described. For example, quantitative polymerase chain reaction (qPCR) enables both detection and quantification of DNA (Lin et al., 2000). Due to its superior sensitivity compared to other PCR assays, a qPCR targeting a 529-bp repeat element is considered the preferred assay for detecting *T. gondii* (Su et al., 2010). Molecular screening can detect the DNA of *T. gondii* stages present in biological samples with high specificity (Liu et al., 2015; Su et al., 2010) which can be followed by molecular characterisation of the pathogen.

Phylogenetic studies based on human and animal isolates have identified three main clonal lineages of *T. gondii*: type I, type II and type III (Ajzenberg et al., 2004; Howe and Sibley, 1995). These predominant clonal lineages account for most *T. gondii* infections in humans and domestic animals in Northern Hemisphere (Galal et al., 2019), however recent population genetic studies have revealed more diverse genotypes in other parts of the world, particularly in Central and South America (Rajendran et al., 2012; Shwab et al., 2014). The presence of both sexual and asexual reproduction phases may lead to a high genetic diversity of *T. gondii*, resulting in both atypical and typical isolates (Grigg and Sundar, 2009). High genetic diversity is suggested to exist among sylvatic cycles in wildlife species (Dardé, 2008; Wendte et al., 2011); for example, most infections with *T. gondii* in California sea otters (*Enhydra lutris nereis*) have been shown to be caused by a novel genotype “X” (Miller et al., 2004). The virulence of *T. gondii* infection and clinical outcomes in intermediate hosts can be related to the associated genotype (Dubey and Jones, 2008; Su and Dubey, 2020). However, genotype-associated virulence may vary among host species. Type I and type I-like strains are associated with ocular and congenital diseases in humans, and type X appears to be fatal in marine mammals (Grigg et al., 2001; Miller et al., 2004; Saeij et al., 2005). In Australia, characterisation of *T. gondii* infecting marsupial species has been largely limited to macropods. Non-archetypal type II-like and genetically diverse atypical strains were reported in several macropod species (Pan et al., 2012; Parameswaran et al., 2010). However, despite advancements in genotyping, limited information is available on the genetic diversity of *T. gondii* strains that infect other Australian marsupials. Most importantly, it is still unclear how, and if, *T. gondii* genotype contributes to the variation in susceptibility observed in marsupial species. This knowledge gap becomes particularly pertinent in endangered marsupial species such as the eastern barred bandicoot.

This study aimed to identify *T. gondii* genotypes found in eastern barred bandicoots in Victoria; this was achieved by screening opportunistically collected tissues of eastern barred bandicoots for the presence of *T. gondii* DNA and subsequent genotype analysis. The findings of this study elucidate the risk of infection with *T. gondii* and provide insights into genotype-associated virulence in introduced eastern barred bandicoot populations, indicating a potential conservation threat.

## 2 Materials and methods

### 2.1 Animals, location and samples

**Fig. 1:**
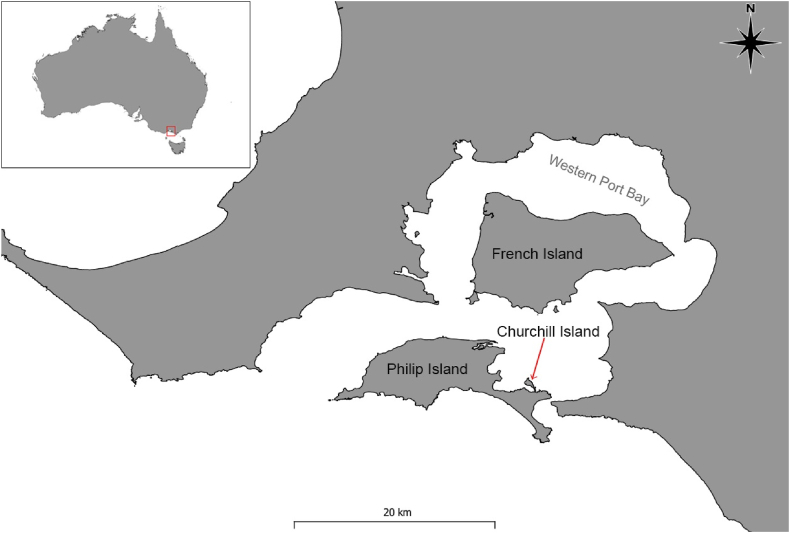
Location of Phillip, French and Churchill Islands in Western Port Bay, Victoria, where eastern barred bandicoot (*Perameles gunnii*) carcasses were collected during 2017–2022. The inset shows the Australian map highlighting the location of study areas.

Samples for this study were opportunistically obtained as part of a more extensive epidemiological study of the impacts of toxoplasmosis on the establishment of populations of eastern barred bandicoots on three islands in southern Victoria (Lynch et al., 2025). Phillip Island (38.4899° S, 145.2038° E), French Island (38.3489° S, 145.3365° E) and Churchill Island (38.4992° S, 145.3379° E) are three adjacent islands located in Western Port (Warn Marin), Victoria, Australia, with an approximate area of 10,000 ha, 18,000 ha and 57 ha, respectively (DCCEEW, 2019) (Fig. 1).

Tissues from a total of 113 eastern barred bandicoots found dead were collected during necropsy by Zoos Victoria between 2017 and 2022. Pathological investigations, including necropsy, were conducted to determine the possible cause(s) of death in bandicoot carcasses collected (for details see Lynch et al., 2025). Veterinary staff at Zoos Victoria performed necropsies and tissue collection. Eastern barred bandicoot carcasses were defrosted at room temperature before necropsies were performed. New scalpel blades and thoroughly cleaned instruments were used between each necropsy to prevent cross-contamination between carcasses. From each carcass, a total of 5 g of tissues, consisting of 2 g of brain and 1 g each of heart, tongue, and diaphragm were collected into 20 ml conical tubes. Tissues were then stored at −20 °C and transported to the Melbourne Veterinary School for further processing.

### 2.2 DNA extraction

Tissue digestion and DNA extraction were performed following protocols described previously (Adriaanse et al., 2020) with minor modifications. Briefly, Tris-EDTA lysis buffer (40 mM Tris, 10 mM EDTA) was added to 5 g of pooled tissues to make up a final volume of 10 ml and incubated in a water bath for 10 min at 90 °C. Subsequently, samples were cooled until room temperature and 200 μl of proteinase K (Promega, Australia) was added and incubated overnight in a water bath at 55 °C. Following a thorough homogenisation using a mechanical tissue homogeniser (POLYTRON® PT 2500 E, Kinematica, USA), DNA was extracted from the tissue lysate using DNeasy Blood & Tissue Kit (QIAGEN, Germany). Subsequently, 300 μl of tissue lysate was added into a 1.5 ml microcentrifuge tube and 300 μl of tissue lysis buffer (Qiagen Buffer AL) was added before incubation at 70 °C for 10 min. Then, samples were centrifuged at 14,000 rpm for 1 min, and 380 μl supernatant was removed into a new microcentrifuge tube containing 200 μl ethanol (96–100 % molecular biology grade ethanol). Subsequently, 580 μl of lysate suspension was transferred into a spin column placed inside a clean collection tube and centrifuged at 8000 rpm for 1 min. Then, the spin column was placed inside a new collection tube and 500 μl of wash buffer (QIAGEN buffer AW1) was added before the centrifugation at 8000 rpm for 1 min. After this, the spin column was placed inside a new collection tube and 500 μl wash buffer (QIAGEN buffer AW2) was added before centrifugation at 14,000 rpm for 3 min. Finally, a spin column was placed inside a labelled micro-centrifuge tube and DNA was eluted by adding 200 μl elution buffer (QIAGEN buffer AE) followed by centrifugation at 8000 rpm for 1 min. Nucleic acid concentration and purity of the extracted DNA samples were measured using a spectrophotometer (NanoDrop™ 2000, ThermoFisher Scientific).

### 2.3 Quantitative polymerase chain reaction (q PCR)

Detection of *T. gondii* DNA in the extracted genomic DNA samples was carried out using TaqMan probe-based qPCR assay (Lelu et al., 2011) targeting 529-bp repeat element (GenBank: AF487550.1) of *T. gondii* (Reischl et al., 2003). Equine Herpes Virus (EHV) DNA (synthetic DNA) was added to the reaction mixture as an internal control to detect any PCR inhibition (Adriaanse et al., 2020). Integrated DNA Technologies, Singapore synthesised all primers and probes (*T. gondii* 529-bp repeat element TaqMan probe: 5'-/56-FAM/ACGCTTTCC/ZEN/TCGTGGTGAT GGC G/3IABkFQ/-'3 and primers 5′-AGAGACACCGGAATGCGATCT-'3 and 5′-CCCTCTTCTCCACTCTTCAATTCT-'3; EHV TaqMan probe 5'-/5HEX/TTTCGCGTG/ZEN/CCTCCTCCAG/3IABkFQ/-'3 and primers 5′-GATGACACTAGCGACTTCGA-'3 and 5′-CAGGGCAGAAACCATAGACA-'3.) and synthetic DNA. The final amplification mixture (20 μl) consisted of 10 μl of GoTaq® Probe qPCR Master Mix (A610A, Promega), 150 nM of each *T. gondii* primer, 75 nM of *T. gondii* TaqMan probe, 40 nM of each EHV primer, 50 nM of EHV TaqMan probe, 1 μl of EHV template DNA and 2 μl of sample template DNA. The qPCR was performed using Rotor-Gene Q real-time PCR Cycler (QIAGEN) under initial denaturation at 95 °C for 1 min followed by 40 cycles of denaturation at 95 °C for 15 s and annealing/extension at 60 °C for 1 min. Positive (*T. gondii* synthetic DNA) and negative controls were included in each run and samples were run in duplicates. The cut-off Cq value used in this study was 33 and it was determined using a standard curve of known *T. gondii* concentrations starting from 500 pg/μl to 5 × 10^−8^ pg/μl, where target DNA was consistently detected at a concentration of 5 × 10^−7^ pg/μl at a Cq value of 33.

### 2.4 Mutilplex multilocus nested PCR and DNA sequencing

A multiplex PCR was carried out for qPCR positive bandicoot DNA samples with molecular markers, including 3′SAG2, 5′SAG2, GRA6, c22–8 and L358 (see Table 1 for primers) following established protocols (Su et al., 2010) with some modifications. PCRs were performed in a thermocycler (T100 Thermal Cycler, Bio-Rad). The final volume of the PCR mixture was 25 μl, containing 2.5 μl GoTaq® Flexi Buffer (M891A, Promega), 2.5 mM MgCl_2_ (A351B, Promega), 160 nM of each external forward and reverse primers, 200 μM each dNTPs (U151A, Promega), 1 unit of GoTaq® Flexi DNA Polymerase (M829A, Promega) and 1.5 μl of each DNA sample. The reaction mixture was denatured at 95 °C for 4 min followed by 30 cycles of 94 °C for 30 s, 55 °C for 1 min, 72 °C for 2 min followed by a final extension at 72 °C for 5 min. Then amplified multiplex PCR products were diluted 1:1 with UltraPure™ DNase/RNase-Free Distilled Water (#10977015, Thermo Fisher Scientific). The nested PCR reaction was carried out individually for each molecular marker in a final volume of 25 μl containing 2.5 μl GoTaq® Flexi Buffer (M891A, Promega), 2.5 mM MgCl2 (A351B, Promega), 300 nM of each nested forward and reverse primer for each genetic marker, 200 μM each dNTPs (U151A, Promega), 1 unit of GoTaq® Flexi DNA Polymerase (M829A, Promega) and 1.5 μl of each diluted multiplex PCR product. The reaction mixture was treated at 95 °C for 4 min followed by 35 cycles of 94 °C for 30 s, 60 °C for 1 min, and 72 °C for 1.5 min followed by a final extension at 72 °C for 5 min. Similarly, *T. gondii* reference DNA representing genotypes I (RH), II (ME49), III (CTG) were also amplified as described above. All amplified nested PCR products were visually assessed for quality using 1.5 % agarose gels stained with GelRed nucleic acid gel stain (#41003, Biotium, Australia) before they were bi-directionally sequenced by Macrogen Inc. (Seoul, South Korea) using PCR primers in separate reactions. The quality of the sequences was appraised using Geneious Prime 2023.2.1. Software (www.geneious.com).

**Table 1:** Molecular markers, multiplex and nested polymerase chain reaction primers (PCR) used to characterise *Toxoplasma gondii* detected in eastern barred bandicoot (*Perameles gunnii*) populations in Victoria, Australia.

Molecular marker	Primer name	Multiplex PCR primers	Nested PCR primers	Nested PCR product size (bp)
L358	L358 - Forward	TCTCTCGACTTCGCCTCTTC	AGGAGGCGTAGCGCAAGT	418
	L358 - Reverse	GCAATTTCCTCGAAGACAGG	CCCTCTGGCTGCAGTGCT	
c22-8	c22-8 - Forward	TGATGCATCCATGCGTTTAT	TCTCTCTACGTGGACGCC	521
	c22-8 - Reverse	CCTCCACTTCTTCGGTCTCA	AGGTGCTTGGATATTCGC	
5′-SAG2	5′-SAG2 - Forward	GCTACCTCGAACAGGAACAC	GAAATGTTTCAGGTTGCTGC	242
	5′-SAG2 - Reverse	GCATCAACAGTCTTCGTTGC	GCAAGAGCGAACTTGAACAC	
3′-SAG2	3′-SAG2 - Forward	TCTGTTCTCCGAAGTGACTCC	ATTCTCATGCCTCCGCTTC	222
	3′-SAG2 - Reverse	TCAAAGCGTGCATTATCGC	AACGTTTCACGAAGGCACAC	
GRA6	GRA6 - Forward	ATTTGTGTTTCCGAGCAGGT	TTTCCGAGCAGGTGACCT	344
	GRA6 - Reverse	GCACCTTCGCTTGTGGTT	TCGCCGAAGAGTTGACATAG	

### 2.5 Virtual restriction fragment length polymorphism (RFLP) and genotyping

DNA sequences of the *T. gondii* amplified for each molecular marker were subjected to genotyping by virtual RFLP using NEBcutter (Version v3.0.17, New England BioLabs Inc. (https://nc3.neb.com/NEBcutter/) and compared with sequences of reference genotypes I (RH), II (ME49), III (CTG) as previously described by Donahoe et al. (2014).

## 3 Results

Overall, 19.5 % (22/113) of tested eastern barred bandicoot carcasses collected were positive for *T. gondii* using qPCR. Out of the 22 qPCR positive samples, only 11 isolates were successfully amplified for all five genetic markers using nested PCR. Most of these amplified isolates (*n* = 10) had a Cq value < 25 in qPCR (data not shown).

Sequence-based RFLP was performed for all 11 *T. gondii* positive isolates from eastern barred bandicoots and compared with those of reference *T. gondii* genotypes. Based on virtual RFLP gel images for each genetic marker (3′SAG2, 5′SAG2, GRA6, c22–8, L358) (Supplementary Fig. 1), all eastern barred bandicoot isolates were similar to *T. gondii* type II (Table 2). However, two eastern barred bandicoots (EBB 1 & EBB 38) revealed unique, single nucleotide polymorphisms (SNPs) at nucleotide position 97 at the c22-8 locus (Table 3) which represent previously undescribed alleles. Therefore, these two *T. gondii* genotypes were identified as type-II-like (Table 2). Both SNPs were substitutions instead of deletions or insertions. However, these SNPs did not interfere with the restriction enzyme cleavage sites, and therefore, a classic type II banding pattern was observed in the virtual RFLP gel image of c22-8 (Supplementary Fig. 1).

**Table 2:** Genotypes of *Toxoplasma gondii* detected in introduced eastern barred bandicoot (*Perameles gunnii*) populations in Victoria, Australia.

Genotype/Sample details	3′-SAG2	5′-SAG2	GRA6	c22-8	L358	Genotype
Reference						
Type I (RH)	I/III	I/II	I	I	I	Type I
Type II (ME49)	II	I/II	II	II	II	Type II
Type III (CTG)	I/III	III	III	III	III	Type III
Eastern barred bandicoot (EBB)						
EBB 1	II	I/II	nd	U-1^a^	II	Type II-like
EBB 4	II	nd	nd	II	II	Type II
EBB 8	II	I/II	II	II	II	Type II
EBB 20	nd	nd	II	II	II	Type II
EBB 24	II	I/II	II	II	II	Type II
EBB 27	II	I/II	II	II	II	Type II
EBB 30	nd	I/II	II	II	II	Type II
EBB 31	II	I/II	II	nd	II	Type II
EBB 36	nd	I/II	II	II	II	Type II
EBB 38	nd	nd	II	U-1^a^	II	Type II-like
EBB 40	II	I/II	II	II	II	Type II

nd = no data; ^a^c22-8 is a unique genotype with single nucleotide polymorphism (SNP) at nucleotide 97 position as shown in Table 3. These two SNPs did not interfere with the classic type II banding pattern in the virtual RFLP gel shown in Supplementary Fig. 1.

**Table 3:** Single nucleotide polymorphisms (SNPs) identified at the c22-8 locus from the *Toxoplasma gondii* detected in eastern barred bandicoot (*Perameles gunnii*) populations in Victoria, Australia.

Locus	c22-8					
Nucleotide (bp number)	41	71	97	143	351	Allele
Consensus	A/T	A/G	C/G	C/T	A/G	
Type I (RH)	A	A	C	C	A	I
Type II (ME49)	.	.	.	T	G	II
Type III (CTG)	T	G	.	.	.	III
EBB 1	A	A	G	.	.	U-1^a^
EBB 4	.	.	C	.	.	II
EBB 8	.	.	.	.	.	II
EBB 20	.	.	.	.	.	II
EBB 24	.	.	.	.	.	II
EBB 27	.	.	.	.	.	II
EBB 30	.	.	.	.	.	II
EBB 31	.	.	.	.	.	II
EBB 36	.	.	.	.	.	II
EBB 38	.	.	G	.	.	U-1^a^
EBB 40	.	.	.	.	.	II

EBB = eastern barred bandicoot.

a These alleles were unique to this study.

## 4 Discussion

This study successfully detected and characterised *T. gondii* in the tissues of eastern barred bandicoots, part of an assisted colonisation program on islands in Victoria, Australia. Genotypes were characterised using DNA sequences of five markers by employing RFLP. To the authors’ knowledge, this is the first time the molecular characterisation of *T. gondii* has been reported from free-ranging eastern barred bandicoots, one of the marsupial species that appear to be most susceptible to clinical toxoplasmosis.

Overall, *T. gondii* was detected in nearly 20 % (22/113) of the carcasses collected. The qPCR assay targeting 529-bp repeat element used in this study is thought to be 10–100 times more sensitive than conventional PCR assays targeting the B1 gene (Pan et al., 2012; Parameswaran et al., 2009; Su et al., 2010), making it the preferred target for the detection of *T. gondii* DNA in biological samples (Homan et al., 2000; Reischl et al., 2003; Su et al., 2010). Previously, Adriaanse et al. (2023), who employed a similar qPCR to that used in the present study, did not find evidence of *T. gondii* in the tissues of any of the eastern barred bandicoot carcasses recovered during a 500-day post-introduction health monitoring programme. However, that study had a much smaller sample size (*n* = 8), with only 67 bandicoots on Phillip Island at the time.

Genotyping analysis revealed most eastern barred bandicoots were infected with type II genotype of *T. gondii*, whereas two animals were infected with type II-like genotypes with unique novel single nucleotide polymorphisms (SNPs) in c22-8 locus. However, these unique SNPs did not change the pattern of bands on the virtual RFLP gel (see Supplementary Fig. 1, c22-8). This highlights the effectiveness of the DNA sequence-based virtual RFLP genotyping approach in detecting SNPs, contributing to an overall better understanding of *T. gondii* genetic diversity (Brennan et al., 2016). Clonal type II is the most predominant genotype causing *T. gondii* infections in humans and animals globally (Fazel et al., 2021; Fernández-Escobar et al., 2022) and has previously been demonstrated in Australian animals, including domestic cats (*Felis catus*) from Sydney, some of which were co-infected with type II-like strains with unique novel SNPs at 3′-SAG2 and L358 loci (Brennan et al., 2016). Globally, atypical strains are predominant in cats followed by clonal type II strains (Amouei et al., 2020). Similarly, *T. gondii* type II and type II-like strains were found in a dog (*Canis familiaris*) and New Zealand fur seal (*Arctocephalus forsteri*) isolates, respectively, from Australia (Al-Qassab et al., 2009; Donahoe et al., 2014). In contrast, a dominance of non-archetypal type II and atypical strains have been reported in other marsupial species, especially macropods (Pan et al., 2012; Parameswaran et al., 2010). For example, Pan et al. (2012) reported genetically distinct multiple *T. gondii* recombinant genotypes in different organs of three species of sub clinically infected macropods in Western Australia. Similarly, predominantly non-archetypal type II genotypes were reported in sub clinically infected western grey kangaroos (Parameswaran et al., 2010). It has been suggested that the high diversity of *T. gondii* genotypes described from macropods may be due to the mixing of clonal lineages as a result of regular sexual reproduction cycles in cats combined with outcrossing with wild intermediate hosts and self-fertilisation (Ajzenberg et al., 2004; Grigg and Sundar, 2009; Pan et al., 2012). The extent of self-fertilisation and outcrossing is influenced by the rate of concurrent multiple infections in the intermediate hosts present in the area and eventually infecting definitive hosts, resulting in genetically distinct organisms (Pan et al., 2012; Saeij et al., 2005). Based on existing information on species susceptibility to toxoplasmosis, it seems plausible that at least some macropod species survive primary infection with *T. gondii* to be infected by another strain of the same parasite (Pan et al., 2012; Parameswaran et al., 2010). On the other hand, eastern barred bandicoots often die following primary exposure (Bettiol et al., 2000a; Obendorf et al., 1996). Therefore, with the limited possibilities for genetic variation, *T. gondii* type II genotype may remain predominant in these introduced bandicoot populations. Furthermore, small and closed island ecosystems may also restrict the potential of *T. gondii* genetic variation. However, the extent of potential contribution to *T. gondii* genetic diversity by other intermediate hosts on these islands are not known. Therefore, genotypes present in cats and other intermediate hosts need to be evaluated to fully understand the *T. gondii* genetic diversity in the island ecosystems included in this study.

The fact that most animals in this study were killed through motor vehicle trauma (Lynch et al., 2025), means that the levels of virulence associated with the detected genotypes in this species are somewhat unclear. Nonetheless, histopathological examination found fulminating toxoplasmosis in at least four of the type II infected bandicoots (Lynch et al., 2025), suggesting that severe clinical signs, including death, can be associated with this genotype in this host species. It is known that genotype may influence the severity of clinical signs in infected host species (Fernández-Escobar et al., 2021; Saraf et al., 2017). For example, clonal type I is highly virulent and lethal in mice, while type II and III tend to be less virulent and more likely to cause chronic infections (Sibley and Boothroyd, 1992; Su et al., 2002). However, virulence is not consistent between intermediate hosts, and certain genotypes or strains can be lethal to one species while being associated with less severe clinical outcomes in other hosts (Parameswaran et al., 2010; Saeij et al., 2005). Likewise, non-archetypal type II-like strains appear to be non-virulent to macropods (Parameswaran et al., 2010), but have been reported to be fatal in free-ranging bare-nosed wombats (Donahoe et al., 2015). Acute generalised toxoplasmosis was reported in eastern barred bandicoots experimentally fed with type III oocysts, with all infected animals dying within 20 days post-infection (Bettiol et al., 2000a). In another experiment, bandicoots experimentally fed with paratenic hosts (earthworms) carrying type III oocysts were reported to exhibit abnormal behaviours and died within 14 days of infection (Bettiol et al., 2000b). Combining these results with those from the present study, it seems likely that both type II and type III may result in severe toxoplasmosis in eastern barred bandicoots, thereby indicating an overall high susceptibility of the species to *T. gondii* (Obendorf and Munday, 1990).

Aside from the acute health impacts of toxoplasmosis, parasite-mediated behavioural changes such as increased boldness and loss of neophobia (Gering et al., 2021; Ingram et al., 2013) have been observed in intermediate hosts. These behavioural modifications could further increase the likelihood of misadventure-related death. Neurological disease has previously been reported in free-ranging wombats with *T. gondii* type II-like genotypes (Donahoe et al., 2015) and has been linked to an increased chance of being hit by motor vehicles or being predated upon (Hollings et al., 2013). Further studies are warranted and may including experimental infections, to determine the range of impacts and virulence of *T. gondii* genotype(s) infecting eastern barred bandicoots. The gold standard for assessing impacts of *T. gondii* on eastern barred bandicoots would involve experimental infections of the species in captivity. However, due to both the conservation status of the species and ethical considerations of such an experiment, it may be preferable to explore the possibility of longitudinal field studies, where individual animals can be tracked over time and retrieved upon death.

Genotyping results were only available for half of the total number of qPCR positive animals. Most samples with a Cq value above 25 did not allow for amplification of all genetic markers in the nested PCR. This is most likely due to the superior sensitivity of the qPCR assay compared to conventional PCR assays (Su et al., 2010). It is possible that additional genotypes were present but not detected.

## 5 Conclusion

In conclusion, we employed a qPCR assay followed by genotyping to reveal *T. gondii* type II as the most common among infected bandicoots, while type II-like genotypes were also detected in some animals. Future studies involving the definitive host, live and dead eastern barred bandicoots, and other marsupial species sharing the same habitat and feeding ecology, would provide a more in-depth understanding of *T. gondii* epidemiology, genetic diversity and genotype(s) associated virulency in eastern barred bandicoots living in these island ecosystems. Furthermore, studies into the pathogenesis of toxoplasmosis in eastern barred bandicoots and the role of *T. gondii* as a population regulatory factor in this species are warranted.

## Declaration of competing interest

The authors have declared no conflict of interest.

## CRediT authorship contribution statement

**K.L.D. Tharaka D. Liyanage:** Writing – review & editing, Writing – original draft, Visualization, Validation, Software, Methodology, Investigation, Formal analysis, Data curation, Conceptualization. **Michael Lynch:** Writing – review & editing, Resources, Conceptualization. **Oluwadamilola S. Omotainse:** Writing – review & editing, Methodology. **Chunlei Su:** Writing – review & editing, Resources, Methodology. **Jasmin Hufschmid:** Writing – review & editing, Validation, Supervision, Resources, Project administration, Funding acquisition, Formal analysis, Data curation, Conceptualization. **Abdul Jabbar:** Writing – review & editing, Validation, Supervision, Software, Resources, Project administration, Methodology, Formal analysis, Data curation, Conceptualization.

## Ethics declaration

Sample collection for this study was performed with the approval of the Phillip Island Nature Parks Animal Ethics Committee (Ethics No: 22020) and Zoos Victoria Research & Animal Ethics Committee (Ethics No: ZV21010), and work was conducted with a Department of Environment, Land, Water and Planning (DELWP) Wildlife Research Permit (Permit No: 10010080).

## References

[bib1] Adriaanse K., Firestone S.M., Lynch M., Rendall A.R., Sutherland D.R., Hufschmid J., Traub R. (2020). Comparison of the modified agglutination test and real-time PCR for detection of *Toxoplasma gondii* exposure in feral cats from Phillip Island, Australia, and risk factors associated with infection. Int. J. Parasitol Parasites Wildl..

[bib2] Adriaanse K., Lynch M., Sutherland D., Traub R., Lowe J., Hufschmid J. (2023). *Toxoplasma gondii* does not inhibit the assisted colonization of eastern barred bandicoots (*Perameles gunnii*) to Phillip Island, Victoria, Australia. J. Wildl. Dis..

[bib3] Ajzenberg D., Bañuls A.L., Su C., Dumètre A., Demar M., Carme B., Dardé M.L. (2004). Genetic diversity, clonality and sexuality in *Toxoplasma gondii*. Int. J. Parasitol..

[bib4] Al-Qassab S., Reichel M.P., Su C., Jenkins D., Hall C., Windsor P.A., Dubey J.P., Ellis J. (2009). Isolation of *Toxoplasma gondii* from the brain of a dog in Australia and its biological and molecular characterization. Vet. Parasitol..

[bib5] Amouei A., Sarvi S., Sharif M., Aghayan S.A., Javidnia J., Mizani A., Moosazadeh M., Shams N., Hosseini S.A., Hosseininejad Z., Nayeri Chegeni T., Badali H., Daryani A. (2020). A systematic review of *Toxoplasma gondii* genotypes and feline: geographical distribution trends. Transbound. Emerg. Dis..

[bib6] Bettiol S.S., Obendorf D.L., Nowarkowski M., Goldsmid J.M. (2000). Pathology of experimental toxoplasmosis in eastern barred bandicoots in Tasmania. J. Wildl. Dis..

[bib7] Bettiol S.S., Obendorf D.L., Nowarkowski M., Milstein T., Goldsmid J.M. (2000). Earthworms as paratenic hosts of toxoplasmosis in eastern barred bandicoots in Tasmania. J. Wildl. Dis..

[bib8] Brennan A., Donahoe S.L., Beatty J.A., Belov K., Lindsay S., Briscoe K.A., Šlapeta J., Barrs V.R. (2016). Comparison of genotypes of *Toxoplasma gondii* in domestic cats from Australia with latent infection or clinical toxoplasmosis. Vet. Parasitol..

[bib9] Chomel B.B., Belotto A., Meslin F.-X. (2007). Wildlife, exotic pets, and emerging zoonoses. Emerg. Infect. Dis..

[bib10] Cook C.N., Morgan D.G., Marshall D.J. (2010). Reevaluating suitable habitat for reintroductions: lessons learnt from the eastern barred bandicoot recovery program. Anim. Conserv..

[bib11] Dardé M.L. (2008). *Toxoplasma gondii*, “new” genotypes and virulence. Parasite.

[bib12] DCCEEW (2019). National Environmental Science Program Threatened Species Research Hub.

[bib13] de Milliano J., Stefano J., Courtney P., Temple-Smith P., Coulson G. (2016). Soft-release versus hard-release for reintroduction of an endangered species: an experimental comparison using eastern barred bandicoots (*Perameles gunnii*). Wildl. Res..

[bib14] Dickman C.R. (1996). Impact of exotic generalist predators on the native fauna of Australia. Wildl. Biol..

[bib15] Donahoe S.L., Rose K., Šlapeta J. (2014). Multisystemic toxoplasmosis associated with a type II-like *Toxoplasma gondii* strain in a New Zealand fur seal (*Arctocephalus forsteri*) from New South Wales, Australia. Vet. Parasitol..

[bib16] Donahoe S.L., Šlapeta J., Knowles G., Obendorf D., Peck S., Phalen D.N. (2015). Clinical and pathological features of toxoplasmosis in free-ranging common wombats (*Vombatus ursinus*) with multilocus genotyping of *Toxoplasma gondii* type II-like strains. Parasitol. Int..

[bib17] Dubey J., Jones J. (2008). *Toxoplasma gondii* infection in humans and animals in the United States. Int. J. Parasitol..

[bib18] Dubey J.P. (2004). Toxoplasmosis – a waterborne zoonosis. Vet. Parasitol..

[bib19] Dubey J.P., Murata F.H.A., Cerqueira-Cézar C.K., Kwok O.C.H., Su C., Grigg M.E. (2021). Recent aspects on epidemiology, clinical disease, and genetic diversity of *Toxoplasma gondii* infections in Australasian marsupials. Parasites Vectors.

[bib20] EPBC-Act (1999). Environment protection and biodiversity conservation act 1999. Department of climate change. Energy, the Environment and Water (DCCEEW).

[bib21] Fazel R., Rezanezhad H., Solhjoo K., Kalantari M., Erfanian S., Armand B., Jahromi M.E. (2021). PCR-based detection of *Toxoplasma gondii* from cattle in southern Iran. Comp. Immunol. Microbiol. Infect. Dis..

[bib22] Fernández-Escobar M., Calero-Bernal R., Regidor-Cerrillo J., Vallejo R., Benavides J., Collantes-Fernández E., Ortega-Mora L.M. (2021). In vivo and in vitro models show unexpected degrees of virulence among *Toxoplasma gondii* type II and III isolates from sheep. Vet. Res..

[bib23] Fernández-Escobar M., Schares G., Maksimov P., Joeres M., Ortega-Mora L.M., Calero-Bernal R. (2022). *Toxoplasma gondii* genotyping: a closer look into Europe. Front. Cell. Infect. Microbiol..

[bib24] Frenkel J.K., Dubey J.P., Miller N.L. (1970). *Toxoplasma gondii* in cats: fecal stages identified as coccidian oocysts. Science.

[bib25] Frenkel J.K., Ruiz A., Chinchilla M. (1975). Soil survival of *Toxoplasma* oocysts in Kansas and Costa Rica. Am. J. Trop. Med. Hyg..

[bib26] Galal L., Hamidović A., Dardé M.L., Mercier M. (2019). Diversity of *Toxoplasma gondii* strains at the global level and its determinants. Food Waterborne Parasitol.

[bib27] Gering E., Laubach Z.M., Weber P.S.D., Soboll Hussey G., Lehmann K.D.S., Montgomery T.M., Turner J.W., Perng W., Pioon M.O., Holekamp K.E., Getty T. (2021). *Toxoplasma gondii* infections are associated with costly boldness toward felids in a wild host. Nat. Commun..

[bib28] Grigg M.E., Bonnefoy S., Hehl A.B., Suzuki Y., Boothroyd J.C. (2001). Success and virulence in *Toxoplasma* as the result of sexual recombination between two distinct ancestries. Science.

[bib29] Grigg M.E., Sundar N. (2009). Sexual recombination punctuated by outbreaks and clonal expansions predicts *Toxoplasma gondii* population genetics. Int. J. Parasitol..

[bib30] Hillman A.E., Lymbery A.J., Thompson R.C. (2016). Is *Toxoplasma gondii* a threat to the conservation of free-ranging Australian marsupial populations?. Int. J. Parasitol. Parasit. Wildlife.

[bib31] Hollings T., Jones M., Mooney N., McCallum H. (2013). Wildlife disease ecology in changing landscapes: mesopredator release and toxoplasmosis. Int. J. Parasitol. Parasites Wildl..

[bib32] Homan W.L., Vercammen M., De Braekeleer J., Verschueren H. (2000). Identification of a 200- to 300-fold repetitive 529 bp DNA fragment in *Toxoplasma gondii*, and its use for diagnostic and quantitative PCR. Int. J. Parasitol..

[bib33] Howe D.K., Sibley L.D. (1995). *Toxoplasma gondii* comprises three clonal lineages: correlation of parasite genotype with human disease. J. Infect. Dis..

[bib34] Ingram W.M., Goodrich L.M., Robey E.A., Eisen M.B. (2013). Mice infected with low-virulence strains of *Toxoplasma gondii* lose their innate aversion to cat urine, even after extensive parasite clearance. PLoS One.

[bib35] Innes E.A. (2010). A brief history and overview of *Toxoplasma gondii*. Zoonoses Public Health.

[bib36] Johnson A., Roberts H., Munday B. (1988). Prevalence of *Toxoplasma gondii* antibody in wild macropods. Aust. Vet. J..

[bib38] Lelu M., Gilot-Fromont E., Aubert D., Richaume A., Afonso E., Dupuis E., Gotteland C., Marnef F., Poulle M.L., Dumetre A., Thulliez P., Darde M.L., Villena I. (2011). Development of a sensitive method for *Toxoplasma gondii* oocyst extraction in soil. Vet. Parasitol..

[bib39] Lin M.-H., Chen T.-C., Kuo T.-T., Tseng C.-C., Tseng C.-P. (2000). Real-time PCR for quantitative detection of *Toxoplasma gondii*. J. Clin. Microbiol..

[bib40] Liu Q., Wang Z.-D., Huang S.-Y., Zhu X.-Q. (2015). Diagnosis of toxoplasmosis and typing of *Toxoplasma gondii*. Parasites Vectors.

[bib41] Liyanage K.L.D.T.D., Amery-Gale J., Uboldi A.D., Adriaanse K., Firestone S.M., Tonkin C.J., Jabbar A., Hufschmid J. (2024). Seroprevalence and risk factors for *Toxoplasma gondii* exposure in Australian feral and stray cats using an in-house modified agglutination test. Vet. Parasitol..

[bib42] Lynch M., Liyanage K.L.D.T.D., Stent A., Sutherland D.R., Coetsee A., Adriaanse K., Jabbar A., Hufschmid J. (2025). Assessing the impact of *Toxoplasma gondii* in endangered eastern barred bandicoots (*Perameles gunnii*) on Phillip and French islands. J. Wildl. Dis..

[bib43] Miller M.A., Grigg M.E., Kreuder C., James E.R., Melli A.C., Crosbie P.R., Jessup D.A., Boothroyd J.C., Brownstein D., Conrad P.A. (2004). An unusual genotype of *Toxoplasma gondii* is common in California sea otters (*Enhydra lutris nereis*) and is a cause of mortality. Int. J. Parasitol..

[bib44] Mitchell G.F., Myroniuk P.O., Kingston J.L., Slater G.J., Seebeck J.H. (1992). Zoo contributions to the recovery programme for the eastern barred bandicoot *Perameles gunnii* 1988-1991. Int. Zoo Yearbk..

[bib45] Montoya J., Liesenfeld O. (2004). Toxoplasmosis. Lancet.

[bib46] Obendorf D., Munday B., Seebeck J.H., Brown P.R., Wallis R.L., Kemper C.M. (1990). Perameles Gunnii, in Bandicoots and Bilbies.

[bib47] Obendorf D.L., Statham P., Driessen M. (1996). Detection of agglutinating antibodies to *Toxoplasma gondii* in sera from free-ranging eastern barred bandicoots (*Perameles gunnii*). J. Wildl. Dis..

[bib48] Pan S., Thompson R.C.A., Grigg M.E., Sundar N., Smith A., Lymbery A.J. (2012). Western Australian marsupials are multiply infected with genetically diverse strains of *Toxoplasma gondii*. PLoS One.

[bib49] Parameswaran N., O'Handley R.M., Grigg M.E., Fenwick S.G., Thompson R.C.A. (2009). Seroprevalence of *Toxoplasma gondii* in wild kangaroos using an ELISA. Parasitol. Int..

[bib50] Parameswaran N., Thompson R.C.A., Sundar N., Pan S., Johnson M., Smith N.C., Grigg M.E. (2010). Non-archetypal Type II-like and atypical strains of *Toxoplasma gondii* infecting marsupials of Australia. Int. J. Parasitol..

[bib51] Parrott M.L., Coetsee A.L., Hartnett C.M., Magrath M.J.L. (2017). New hope for the eastern barred bandicoot *Perameles gunnii* after 27 years of recovery effort. Int. Zoo Yearbk..

[bib52] Rajendran C., Su C., Dubey J.P. (2012). Molecular genotyping of *Toxoplasma gondii* from Central and South America revealed high diversity within and between populations. Infect. Genet. Evol..

[bib53] Reischl U., Bretagne S., Krüger D., Ernault P., Costa J.-M. (2003). Comparison of two DNA targets for the diagnosis of Toxoplasmosis by real-time PCR using fluorescence resonance energy transfer hybridization probes. BMC Infect. Dis..

[bib54] Saeij J.P.J., Boyle J.P., Boothroyd J.C. (2005). Differences among the three major strains of *Toxoplasma gondii* and their specific interactions with the infected host. Trends Parasitol..

[bib55] Saraf P., Shwab E.K., Dubey J.P., Su C. (2017). On the determination of *Toxoplasma gondii* virulence in mice. Exp. Parasitol..

[bib56] Seebeck J. (1979). Status of the barred bandicoot, *Perameles gunnii*, in Victoria: with a note on husbandry of a captive colony. Wildl. Res..

[bib57] Shwab E.K., Zhu X.-Q., Majumdar D., Pena H.F.J., Gennari S.M., Dubey J.P., Su C. (2014). Geographical patterns of *Toxoplasma gondii* genetic diversity revealed by multilocus PCR-RFLP genotyping. Parasitology.

[bib58] Sibley L.D., Boothroyd J.C. (1992). Virulent strains of *Toxoplasma gondii* comprise a single clonal lineage. Nature.

[bib59] Su C., Dubey J.P., Tonkin C. (2020).

[bib60] Su C., Howe D.K., Dubey J.P., Ajioka J.W., Sibley L.D. (2002). Identification of quantitative trait loci controlling acute virulence in *Toxoplasma gondii*. Proc. Natl. Acad. Sci..

[bib61] Su C., Shwab E.K., Zhou P., Zhu X.Q., Dubey J.P. (2010). Moving towards an integrated approach to molecular detection and identification of *Toxoplasma gondii*. Parasitology.

[bib62] Tenter A.M., Heckeroth A.R., Weiss L.M. (2000). *Toxoplasma gondii*: from animals to humans. Int. J. Parasitol..

[bib63] Todd C.R., Jenkins S., Bearlin A.R. (2002). Lessons about extinction and translocation: models for eastern barred bandicoots (*Perameles gunnii*) at woodlands historic park, Victoria, Australia. Biol. Conserv..

[bib64] Wang Z.D., Liu H.H., Ma Z.X., Ma H.Y., Li Z.Y., Yang Z.B., Zhu X.Q., Xu B., Wei F., Liu Q. (2017). *Toxoplasma gondii* infection in immunocompromised patients: a systematic review and meta-analysis. Front. Microbiol..

[bib65] Wendte J.M., Gibson A.K., Grigg M.E. (2011). Population genetics of *Toxoplasma gondii*: new perspectives from parasite genotypes in wildlife. Vet. Parasitol..

[bib37] Winnard A.L., Coulson G. (2008). Sixteen years of eastern barred bandicoot *Perameles gunnii* reintroductions in Victoria: a review. Pac. Conserv. Biol..

[bib66] Woinarski J.C.Z., Legge S., Fitzsimons J.A., Traill B.J., Burbidge A.A., Fisher A., Firth R.S.C., Gordon I.J., Griffiths A.D., Johnson C.N., McKenzie N.L., Palmer C., Radford I., Rankmore B., Ritchie E.G., Ward S., Ziembicki M. (2011). The disappearing mammal fauna of northern Australia: context, cause, and response. Conserv. Lett..

