# Supplementary material for: Molecular detection and characterisation of Toxoplasma gondii in eastern barred bandicoots (Perameles gunnii) in Victoria, Australia
Source: Int J Parasitol Parasites Wildl. 2025 Apr 11;27:101071. doi: 10.1016/j.ijppaw.2025.101071 (PMC12019445; doi:10.1016/j.ijppaw.2025.101071)
Supplement: Multimedia component 1 [file mmc1.docx]

**Appendix A. Supplementary data**

**3’-SAG2**


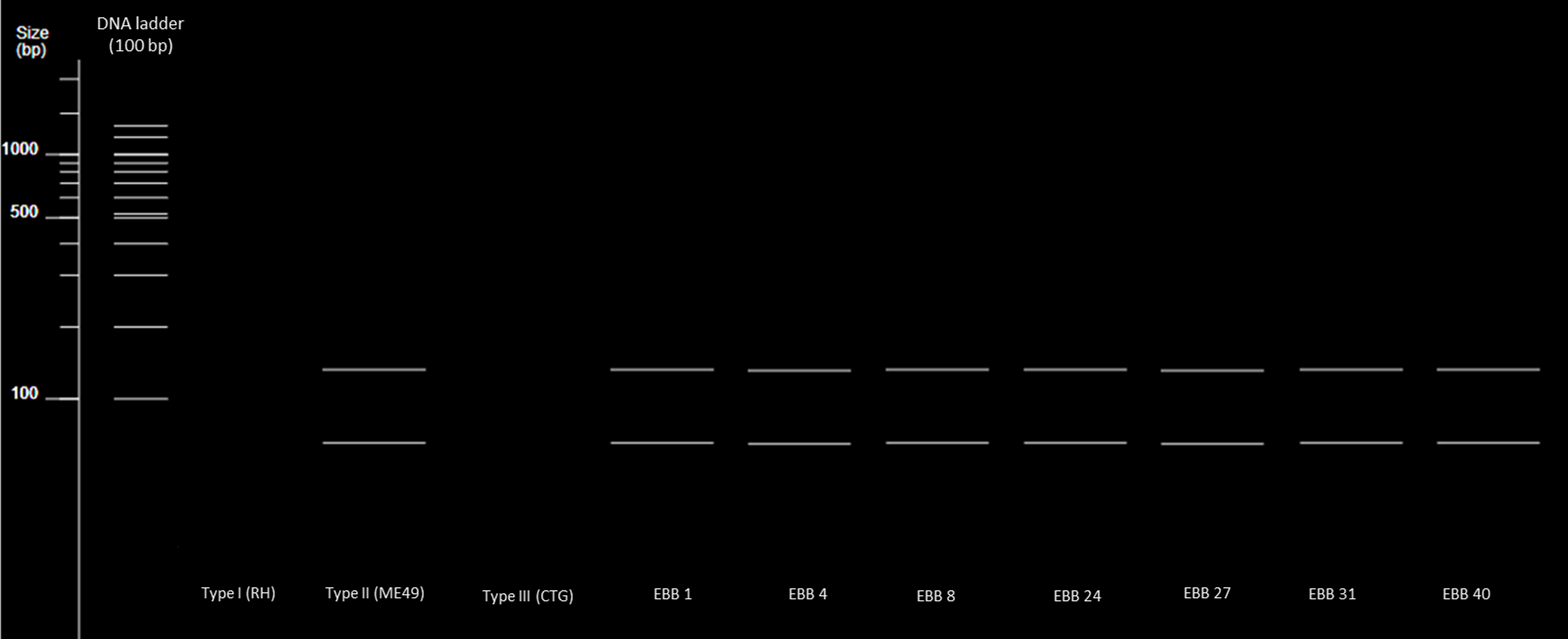


**5’-SAG2**


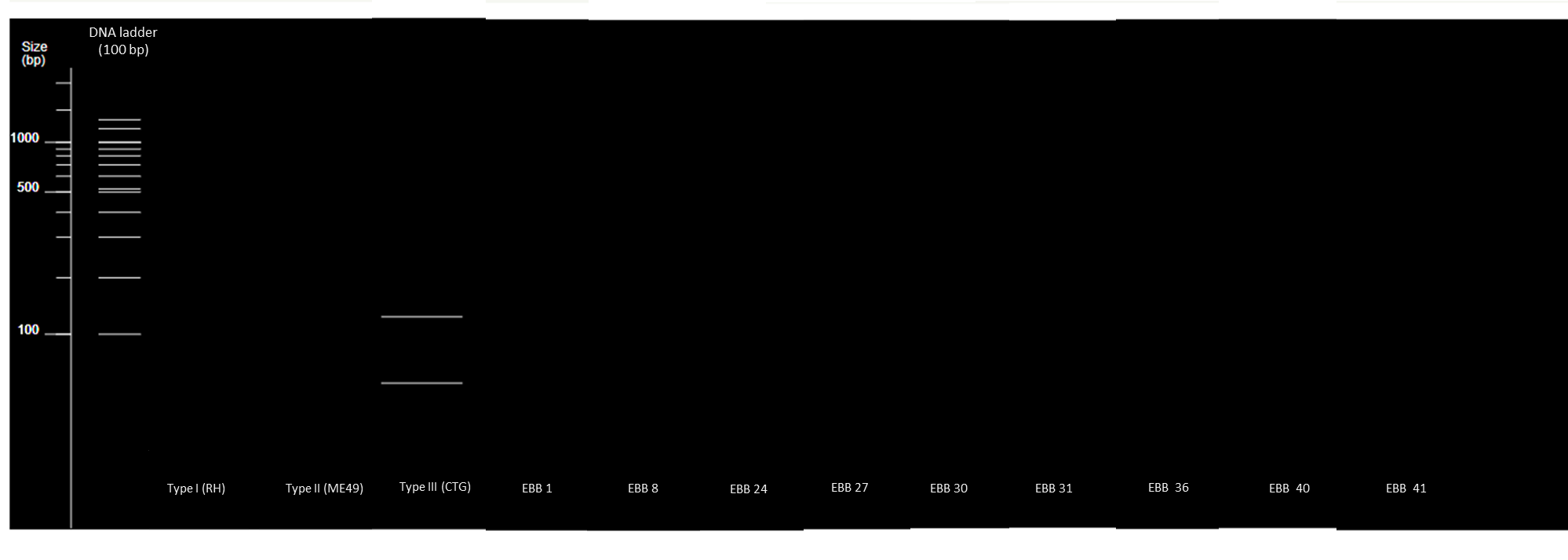


**GRA6**


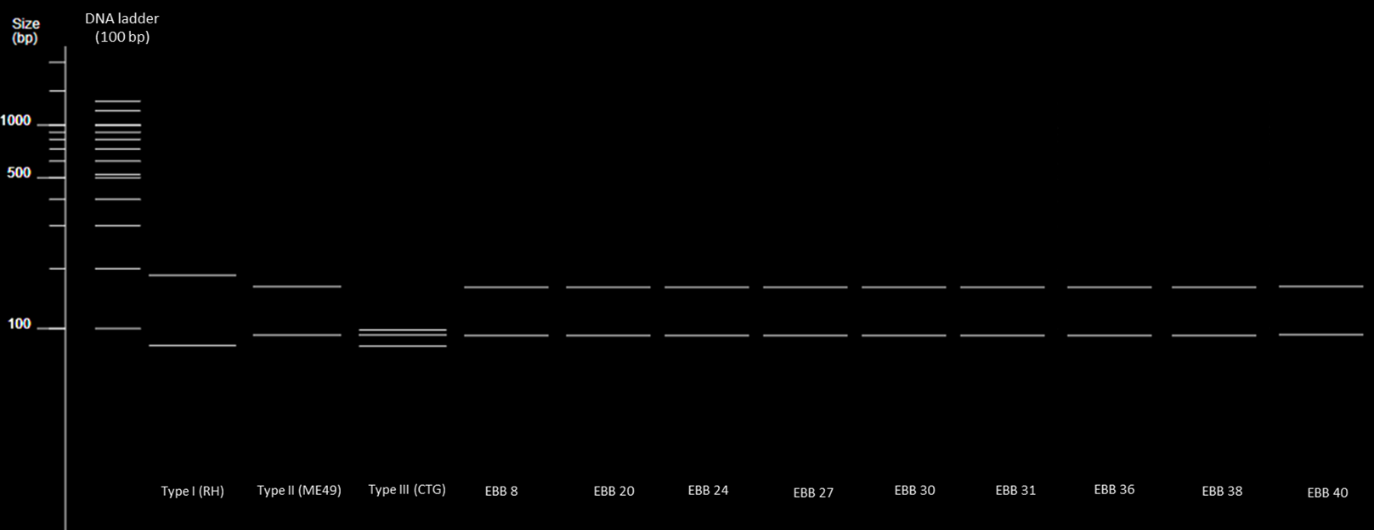


**L358**


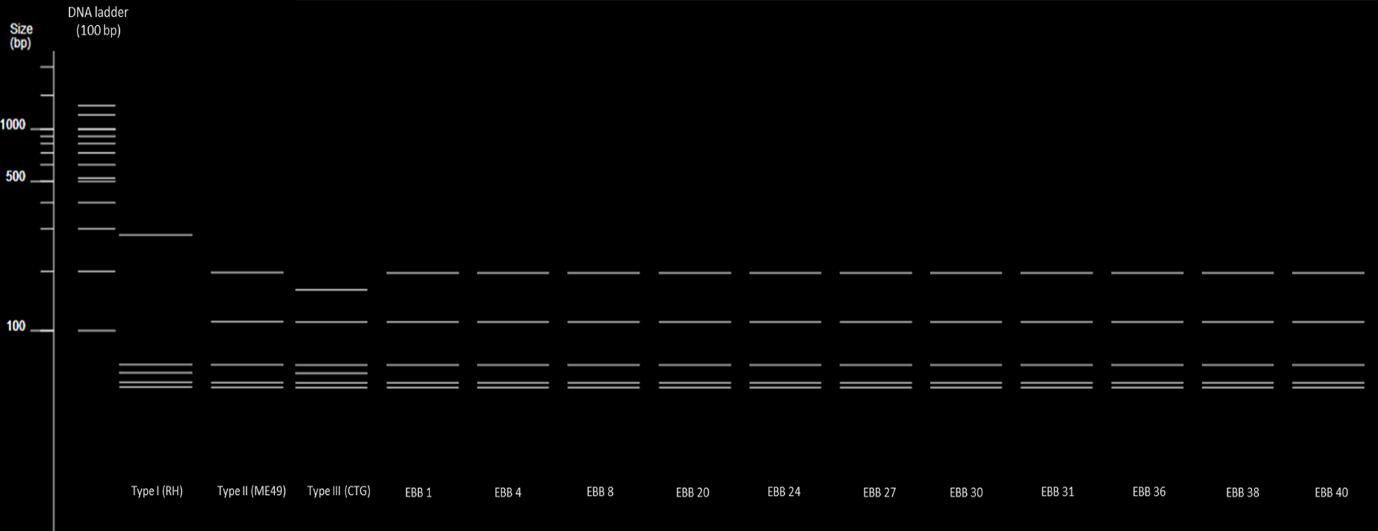


**c22-8**


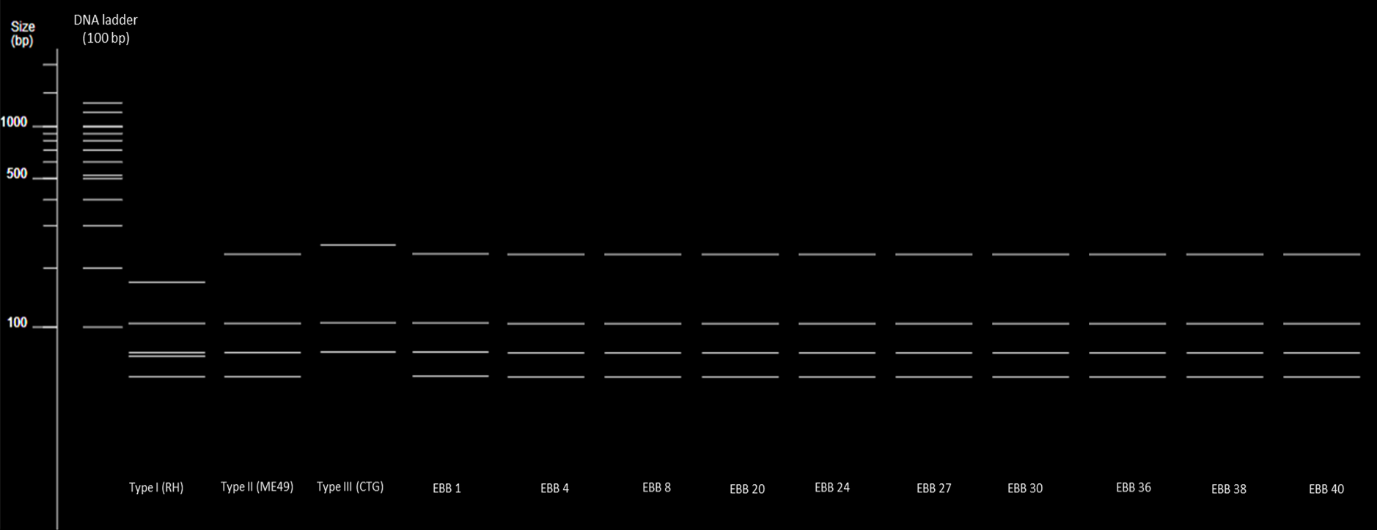


**Supplementary Fig. 1.** DNA sequence-based virtual restriction fragment length polymorphism (RFLP) gel images for each genetic marker, including 3’-SAG2, 5’-SAG2, GRA6, c22-8 & L358 for *Toxoplasma gondii* isolates from eastern barred bandicoots (*Perameles gunnii*). The first column on the left side indicates 100 bp DNA ladder and the next three columns contain RFLP profiles of three reference genotypes (Type I, II, II) for each molecular marker per gel. Virtual RFLP was performed using NEB cutter (https://nc3.neb.com/NEBcutter/) and the gel image for each genetic marker was created by collating the gel images of each relevant genetic marker using Microsoft PowerPoint.
